# Editorial: Colloidal Semiconductor Nanocrystals: Synthesis, Properties, and Applications

**DOI:** 10.3389/fchem.2019.00684

**Published:** 2019-10-22

**Authors:** Vladimir Lesnyak, Maksym Yarema, Shiding Miao

**Affiliations:** ^1^Physical Chemistry, TU Dresden, Dresden, Germany; ^2^Chemistry and Materials Design Group, Department of Information Technology and Electrical Engineering, ETH Zurich, Zurich, Switzerland; ^3^Key Laboratory of Automobile Materials of Ministry of Education, Department of Materials Science and Engineering, Jilin University, Changchun, China

**Keywords:** semiconductor nanocrystals, quantum dots, colloids, perovskite, phosphide, photocatalysis, infrared photodetectors, laser patterning

Colloidal semiconductor nanocrystals (also known as quantum dots) have evolved during the last few decades from purely fundamental concepts to industry-scale commercial products (e.g., Samsung QLED TV displays, in which quantum dots are utilized as color converters). A vivid interest to and rapid development of colloidal nanomaterials is caused by their unique size-dependent optoelectronic properties (based on quantum confinement effects) and by the solution-based synthesis and device fabrication protocols (which are remarkably simple, fast but highly customizable processes).

To date, a large variety of semiconductors are prepared in the form of colloidal nanocrystals, including most-common families of semiconductor materials (i.e., II–VI, IV–VI, III–V, I–III–VI, I–II–IV–VI, and I–IV–VII compounds with corresponding examples being CdSe, PbS, InP, CuInS_2_, Cu_2_ZnSnS_4_, and CsPbBr_3_). Manipulating the reaction conditions (temperature, time, chemistry choice, etc.) provides an accurate control over size and size distribution, morphology, and crystal structure of colloidal nanocrystals. Two or more semiconductors can be combined in one nano-object, resulting in various segmented morphology (e.g., core-shell, dot-in-rod, Janus-type particles, etc.). The surface chemistry of semiconductor colloids can also be tailored via post-synthetic functionalization and ligand exchange surface reactions. Colloidal semiconductor nanocrystals thus become truly interdisciplinary field of science with complex chemistry and physics underlying these apparently simple materials.

In this Research Topic we present a collection of original research and review articles touching upon different aspects of semiconductor nanocrystals, including their synthesis in liquid media (Li et al.) and in solid state via laser patterning (Antolini and Orazi), integration into polymer matrices (Huang et al.; Papagiorgis et al.), applications in photocatalysis, and infrared photodetectors (Livache et al.; Moroz et al.). The Research Topic provides interesting insights into the impact of crystal structure of quantum dots on their fluorescence properties (Ludescher et al.) and into the linking of semiconductor nanocrystals to widely used transparent conductive substrates (Miethe et al.). Materials highlighted in this Topic span from classical CdSe-based structures (Ludescher et al.; Miethe et al.) to nowadays booming lead halide-based perovskite nanocrystals (Huang et al.; Papagiorgis et al.), metal phosphides family (Li et al.; Wegner et al.), and infrared active quantum dots (Livache et al.).

Original research papers of Itskos and Xie groups address well-known problem of insufficient stability of lead halide perovskite nanocrystals, a roadblock toward many industry-scale applications (Huang et al.; Papagiorgis et al.). Papagiorgis et al. employ an electrospinning to produce nanocrystals-in-polymer fibers. The robust composites are prepared either by direct electrospinning mixtures of the nanocrystals in toluene with hydrophobic poly(methyl methacrylate) dissolved in chloroform or by immersion of electrospun hydrophilic polyvinylpyrrolidone membranes into the nanocrystal solutions. Remarkably, the authors achieve a yearlong stability of the prepared composites. Huang et al. propose a facile embedding strategy of nanocrystals into polysterene microparticles (beads) via deliberate control of their swelling in a mixture of solvents. Such nanocrystals-in-polymer composite particles can be tranferred into water, forming stable dispersions with outstanding structural and chemical stability upon prolonged storage time, intense light irradiation, and heat. These approaches help toward successful applications of the perovskite-in-polymer composites as efficient color converters in displays and textile-based flexible light emitting devices (Huang et al.; Papagiorgis et al.).

The work of Lechner group investigates a structural origin of excellent photoluminescence properties of CdSe-CdS core-shell nanocrystals (Ludescher et al.). Using X-ray scattering techniques, the authors retrieve the mean shape and surface of nanocrystals and reveal an elliptical shape with pronounced surface facets for larger nanocrystals related to a mixture of crystal phases within the CdSe core. Correlating the structural and optical data, Ludescher et al. establish the structure-property link between the shape and shell thickness of CdSe-CdS nanocrystals and their photoluminescence efficiency. Dorfs and Bigall groups utilize dot-in-rod CdSe-CdS nanocrystals as an optically addressable probe for the electronic surface states of the transparent indium tin oxide (ITO) conductive glass (Miethe et al.). This study provides the proof of electronic interconnections in ITO-coated glass/linker/nanorod electrodes via easy reproducible functionalization and polishing experiments. Optical characterization reveals changes in the charge carrier dynamics within the system depending on linker molecules.

The contribution of Reiss group concerns a shell engineering of InP-based core-shell nanocrystals, a key Cd-free color converter material nowadays (Wegner et al.). The authors investigate a photostability of InP-based nanocrystals for three different shell compositions: single gradient ZnSe_x_S_1−x_ shell, additional ZnS shell on top of ZnSe_x_S_1−x_, and alumina-coated InP-ZnSe_x_S_1−x_-ZnS nanocrystals. The latter heterostructures exhibit the highest stability upon continuous irradiation with simulated sunlight in a climate chamber.

This Research Topic features several review articles with distinct scopes (Antolini and Orazi; Li et al.; Livache et al.; Moroz et al.). Li et al. discuss metal phosphide nanocrystal colloids. In particular, the authors detail synthetic strategies for InP nanocrystals, highlighting benefits of employing zinc precursors as reaction additives and the importance of different phosphorus precursors to improve the quality of the materials. The authors discuss synthetic approaches for other metal phosphide nanocrystals, such as II–V metal phosphides (Cd_3_P_2_, Zn_3_P_2_) and transition metal phosphides (Cu_3_P, FeP), and summarize their potential applications in photovoltaics, light-emitting diodes, and lithium-ion batteries. Moroz et al. review photocatalytic systems based on colloidal semiconductor nanocrystals coupled to metal catalysts. In such systems, nanocrystal sensitizers demonstrate a compelling performance in homogeneous photoreduction reactions (e.g., the degradation of organic dyes and hydrogen generation), however, the progress beyond half-cycle reactions remains limited. The authors state main challenges and outline perspective directions in the field of photocatalytic applications of nanocrystal colloids (i.e., the possibility of harvesting triplet excitons and utilizing nanocrystal assemblies to accumulate multiple charges at the reaction site). Livache et al. provide a comprehensive view on state-of-the-art nanocrystal-based infrared photodetectors. After a decade of intensive research, infrared colloidal nanocrystals reach high maturity level to be considered as an alternative to the epitaxially-grown infrared semiconductors. The authors highlight the benefits of colloidal nanocrystals, including full tunability of their infrared absorption, reduced production costs, realization of background-limited impurity photoconductor photodiodes, and demonstration of nanocrystal-based focal plane arrays. The authors propose a road map to address remaining challenges in order to promote the infrared nanocrystal-based technology to the industrial level. Antolini and Orazi introduce direct laser patterning approaches to the II-VI semiconductor nanocrystals (e.g., a laser-assisted conversion of precursors) as a simple yet powerful way to produce quantum dots in the solid state. The authors discuss laser parameters (wavelength, pulse duration) and chemistry of precursors to fabricate quantum dot thin film structures with excellent optical properties.

We hope this Research Topic attracts interested readers, providing novel literature insights, synergistic research ideas and enthusiasm in research and studies. Enjoy its reading!

## Author Contributions

All authors listed have made a substantial, direct and intellectual contribution to the work, and approved it for publication.

### Conflict of Interest

The authors declare that the research was conducted in the absence of any commercial or financial relationships that could be construed as a potential conflict of interest.

